# A p16-Ki-67-HMB45 immunohistochemistry scoring system as an ancillary diagnostic tool in the diagnosis of melanoma

**DOI:** 10.1186/s13000-015-0431-9

**Published:** 2015-10-26

**Authors:** Arnaud Uguen, Matthieu Talagas, Sebastian Costa, Sandrine Duigou, Stéphanie Bouvier, Marc De Braekeleer, Pascale Marcorelles

**Affiliations:** Inserm, U1078, Brest, F-29200 France; CHRU Brest, Service d’anatomie et cytologie pathologiques, Brest, F-29220 France; Université Européenne de Bretagne, Rennes, France; Department of Pathology, University Hospital Morvan, 5, Avenue Foch, 29609 Brest, France; CHRU Brest, Laboratoire de Cytogénétique et Biologie de la Reproduction, Brest, F-29220 France

**Keywords:** Melanoma, Nevus, Immunohistochemistry, Ki-67, p16, HMB45

## Abstract

**Background:**

Melanoma is a skin cancer which treatment requires early diagnosis and large surgical removal. The histopathological diagnosis of a melanocytic tumour is sometimes difficult between a benign nevus and a malignant melanoma. We built an immunomarker-based score to differentiate nevi from melanomas.

**Methods:**

Two independent sets of 308 (first set) and 62 (validation set) formalin-fixed and paraffin embedded tumour samples were studied using p16-Ki-67 and HMB45-MelanA dual-staining immunohistochemistry.

**Results:**

In the first set of tumours, high Ki-67 index, low to null p16 immunohistochemistry and absence of HMB45 immunohistochemistry gradient were more frequent in melanomas (156 primary tumours and 78 metastases) than in nevi (74 tumours). Nevertheless, none of these single parameters was able to differentiate all primary melanomas from all nevi. We built a scoring system based on the addition of semi-quantitative scorings of Ki-67 (0: <2 %; 1:2–5 %; 2:6–10 %, 3:11–20 %; 4:>20 %) and p16 (0:>50 % stained cells; 1:11–50 %; 2:1–10 %; 3:0 %) and HMB45 staining (0: gradient present; 1: doubtful/inconclusive gradient; 2: gradient absent). A p16-Ki-67-HMB45 total score from 0 to 9 permitted to classify nevi (score <4) and primary melanomas (score ≥4) with a sensitivity of 97.4 % and a specificity of 97.3 % in the first set of tumours. Sensibility and specificity of 100 % were obtained in a second set (validation set) of 62 tumours (46 melanomas and 16 nevi). The total scoring also allowed analyzing 11 difficult or initially misdiagnosed tumours in our files.

**Conclusions:**

We propose a valuable triple p16-Ki-67-HMB45 immunohistochemistry scoring system to help pathologists in the differential diagnosis of melanomas and nevi.

## Background

Melanoma is a skin cancer with increasing incidence and mortality rates. Surgical removal of the primary lesion before it metastasizes is the main effective therapy, albeit the therapeutic management of metastatic melanoma is now being improved by targeted therapies. Histopathological examination remains currently the “gold standard” for the diagnosis of suspicious pigmented tumours. A definite histopathological distinction between benign and malignant melanocytic tumours may in some cases be difficult. There is also an interobserver variability with differences concerning the diagnosis of nevus versus melanoma in 2.3 to 25 % of tumours [1–5]. Cutaneous melanocytic tumours account for a large proportion of the tissue samples daily studied in pathology departments. In this context, a major reason for a medical malpractice lawsuit in surgical pathology is failure to diagnose melanoma on a skin biopsy [6, 7]. Indeed, failure to recognize melanoma is potentially fatal, but typing a benign lesion as malignant can also lead to morbidity from unwarranted treatment, emotional strain and loss of insurance. In this manner, a test that could help to improve the diagnostic accuracy in the field of melanocytic tumours is needed.

Several markers may be used as diagnostic tools in melanocytic tumours. As nevi and melanomas differ in the absence or presence of chromosomal aberrations respectively, cytogenetic tools such as Comparative Genomic Hybridizations on metaphases (CGH) and on DNA microarray (CGH array) or Fluorescent in situ Hybridization (FISH) have been developed to help pathologists in the classification of ambiguous melanocytic tumours [8–10]. Nevertheless, these molecular methods require expensive equipment and pathologists highly skilled in molecular methods, which are barriers to the general use of these new tools. On the opposite, immunohistochemistry is a more largely diffused and routinely used technique in most of the pathology centers and immunohistochemical markers may be easier to apply than molecular methods. Nevertheless, even if several markers have been studied, only few of them appear as possible tools for distinguishing melanomas from nevi. To date, none of them is enough accurate to distinguish melanomas from nevi. Ki-67 (a nuclear proliferation marker present in two 345 kDa and 395 kDa isoforms and expressed in late G1, S, G2 and M phases but not in G0 phase), p16 (a protein regulating the G1/S checkpoint of the cell cycle and the product of the tumour suppressor gene *CDKN2A*) and HMB45 (an anti-gp100 antibody labelling the cytoplasm of intra-epidermal, “immature” and “activated” melanocytes) are some examples [11–16]. As none of the tested single markers had been evaluated accurate enough to classify a melanocytic lesion as nevus or melanoma, we hypothesize that a combination of these markers can nevertheless help the pathologists to differentiate between benign and malignant melanocytic tumours.

In this study, we propose a triple immunomarker-based score considering p16, Ki-67 and HMB45 to differentiate nevi and melanomas.

## Methods

### Tissue samples

Paraffin-embedded tissues from a first tumour set of 308 tumours were extracted from the archives of the Department of Pathology at the Brest University Hospital and regional collaborators. Based on histopathological reports, these tumours were separated in 74 nevi (13 compound, 10 junctional, 18 dermal, 4 congenital, 11 Spitz, 6 Reed, 1 acral, 1 deep-penetrating, 7 conventional blue and 3 cellular blue), 156 primary melanomas (66 superficial spreading [SSM], 46 nodular, 15 lentigo malignant, 7 acro-lentiginous, 8 mucosae, 5 desmoplastic, 2 nevoid, 1 spizoid and 6 unclassified), and 78 metastases (39 nodal, 27 cutaneous, 3 cerebral, 3 pulmonary, 2 mesenteric, 1 colonic, 1 gastric, 1 renal and 1 adrenal). All cases underwent a histopathological review on Hematoxylin-Eosin-Saffron (HES) stained slides to assess well-preserved tumor tissue and select the most representative tissue block. The tumours were classified as nevi, primary melanomas or metastases according to the histopathological report. Nevertheless, 4 tumours considered as nevi on initial reports were reclassified as melanomas because of metastatic evolution. This first set of tumours was used to build an immunohistochemical scoring system to distinguish benign and malignant tumours.

A second independent file of 62 melanocytic tumours (validation set) was then analyzed with the score established in the first tumour set. All cases also underwent a histopathological review on HES stained slides. One of the tumours was initially mistyped as a SSM and was reclassified as a compound nevus on the basis of the new histopathological examination. A spitzoid lesion required a review by an expert in melanocytic pathology and was finally classed as a spitzoid melanoma. This validation set finally comprised 46 malignant melanomas (27 SSM, 9 nodular, 3 lentigo malignant, 3 acro-lentiginous, 1 spitzoid and 3 unclassified) and 16 nevi (5 compound, 1 junctional, 7 dermal, 1 congenital, 2 Spitz).

All samples were included in a registered tumour tissue collection and the present study was conducted in compliance with the Helsinki Declaration and after approval by our institutional review board (CHRU Brest, CPP n° DC – 2008 – 214).

### Immunohistochemistry

Two double-staining immunohistochemical techniques were performed for each case on 4-μm tissue sections mounted on Superfrost® Plus slides (Thermo Scientific, Saint-Herblain, France) dried overnight at 37 °C before processing. Double stain IHC was performed on Ventana Benchmark XT® automated slide preparation system (Roche Diagnostics, Meylan, France) using two different revelation kits: ultraView Universal DAB Detection Kit (Roche Diagnostics) and ultraView Universal Alkaline Phosphatase Red Detection Kit (Roche Diagnostics). This technique concerned Ki-67 (clone MIB-1, Dako, Glostrup, Denmark, 1:50 dilution, revealed with DAB labelling) and p16 (clone E6H4, Ventana-Roche Diagnostics, prediluted, revealed with Red labelling) on a first slide and, on a second slide, HMB45 (clone HMB45, Dako, 1:50 dilution, revealed with DAB labelling) and MelanA (clone A103, Dako, 1:25 dilution, revealed with Red labelling). The Ki-67/p16 slides underwent a pretreatment with cell conditioner 1 (pH 8) for 30 min, followed by incubation with Ki-67 antibody at 37 °C for 32 min. After washing and an antibody denaturation step at 95 °C for 8 min, the p16 antibody was incubated at 37 °C for 32 min. After a second washing step, the slides underwent counterstaining with one drop of hematoxylin for 12 min and one drop of bluing reagent for 4 min. Subsequently, slides were removed from the immunostainer, washed in water with dishwashing detergent, and mounted. The HMB45/MelanA slides underwent a similar process except for a longer (60 min) pretreatment and a first incubation with HMB45 antibody followed by a second incubation with MelanA antibody (32 min of incubation for each antibody).

### Immunohistochemistry analysis

For each case, both IHC slides were interpreted independently of the diagnosis established on the HES stained slide. Concerning the Ki-67/p16 slide, the Ki-67 index was determined in the most proliferative area without major inflammatory infiltrate on the basis of a low magnification eye-balling. In this “hot spot”, the percentage of nuclear DAB stained cells was estimated in about 200 tumour cells, allowing the interpretation of thin and thick lesions. The percentage of p16 Red-stained cells was estimated in the full tumor section, without differentiating between nuclear and/or cytoplasmic staining. Positive controls for Ki-67 immunostaining consisted in proliferative non tumour cells such as basal keratinocytes.

Considering the HMB45/MelanA slide, we did not quantify the percentage of stained tumour cells. The HMB45 gradient was considered to be positive when only the most superficial cells were HMB45 stained. It was negative when the staining involved equally the superficial and deep parts of the tumour. It was inconclusive/doubtful when no or few cells were stained. HMB45 gradient was only assessed for nevi and primary melanomas. MelanA staining was not quantified but only used as a second melanocytic marker revealing the melanocytic cells and helping to qualify the existence or absence of a HMB45 gradient.

Staining intensity (i.e., weak or strong staining) was not taken into account for any of the markers.

### Fluorescent in situ hybridization

Two tumours were studied using fluorescent in situ hybridization techniques according to previous reported FISH process [17]. We assembled eight Bacterial Artificial Chromosome (BAC) clones from the libraries of the Roswell Park Cancer Institute to prepare four FISH probes sets (Table 1). The probes were validated on normal metaphases. Four 4-μm tissue-slides were hybridized per tumour (4 probes sets) and were scanned and analyzed using a PathScan imaging software (PATHSCAN® FISH, Excilone, Elancourt, France) to enumerate each probe signals searching for chromosomes 6, 8, 9 or 11 imbalances.

**Table 1:** Bacterial Artificial Chromosome (BAC) clones used to prepare FISH probes

BAC clone	Chromosomal locus	Labelling	Probes sets
RP11-61O16	*RREB1* (6p25)	Spectrum Red	N°1
RP11-323 N12	*MYB* (6q23.3)	Spectrum Green	N°1
RP11-1007G14	*HRAS* (11p15.5)	Spectrum Red	N°2
RP11-156B3	*CCND1* (11q13.3)	Spectrum Green	N°2
RP11-440 N18	*C-MYC* (8q24.1)	Spectrum Red	N°3
RP11-1084C20	*POTEA* (8p11.1)	Spectrum Green	N°3
RP11-478 M20	*CDKN2A* (9p21.3)	Spectrum Red	N°4
RP11-959B21	*GNAQ* (9q21.2)	Spectrum Green	N°4

### Statistical analysis

On the basis of Ki-67 index, p16-stained proportions and the appreciation of a HMB45 gradient, we first compared these three single parameters between nevi, primary melanomas and metastases using one-way analysis of variance (ANOVA) for Ki-67 index and p16 labelling and chi-squared test for HMB45 labelling. The areas under the curve (AUC) of the receiver operating characteristic (ROC) curves analysis were also calculated for benign and malignant primary tumours comparison.

Based on this first step, we developed a semi-quantitative scoring system combining the data of each Ki-67, p16 and HMB45 IHC that were rescored using semi-quantitative scales. Scores obtained for each marker were combined to obtain a double p16-Ki-67 total score and a triple p16-Ki-67-HMB45 total score. The AUC of the ROC curves concerning the combined scores were calculated and compared to the single markers scores.

The tumours from the validation set were analyzed to confirm the performances of the scores developed with the first tumour set.

Statistical analyses were performed using MedCalc Statistical Software version 13.2.2 (MedCalc Software bvba, Ostend, Belgium; http://www.medcalc.org; 2014). The level of significance was set at *P* < 0.05.

## Results

### Single Ki-67, p16 and HMB45 appreciations

We first scored Ki-67 proliferative index and p16 expression in the malignant tumours (i.e., metastases and primary melanomas) and in the benign nevi from the first set of tumours. We also looked for an HMB45 immunohistochemical gradient staining in primary melanomas and nevi. The results are summarized in Table 2. Ki-67 proliferative index was higher in malignant melanocytic tumours than in nevi, and higher in metastases than in primary melanomas. A low to null percentage of p16-immunostained tumour cells was more frequent in malignant melanocytic tumours than in nevi. The absence of HMB45 gradient was more frequent in malignant tumours.

**Table 2:** Summary of the results

		Nevi	Primary melanomas	Metastases	*p*-values	AUC [95 % C.I.]
Ki-67 index	Mean [95%C.I.]	1.7 % [1.3–2.1]	21.7 % [18.9–24.3]	29.9 % [24.9–35]	*p* < 0.001	- nevi vs primary melanomas : 0.959 [0.925–0.981] - benign vs malignant tumours (with metastases): 0.966 [0.94–0.983]
p16 (% of stained cells)	Mean [95%C.I.]	60.5 % [54.4–66.7]	24.8 % [18.9–30.7]	16.2 % [9.1–23.3]	*p* < 0.001	- nevi vs primary melanomas : 0.778 [0.719–0.830] - benign vs malignant tumours (with metastases): 0.804 [0.756–0.847]
HMB45 gradient	Yes:	44.6 %	10.3 %	N. A.	*p* < 0.001	0.74 [0.678–0.795]
	Doubtful:	24.3 %	14.7 %			
	No:	31.1 %	75 %			
p16-Ki67 combined score	Score 0:	48.6 %	0 %	0 %	-	0.982 [0.955–0.995]
	Score 1:	33.8 %	0 %	0 %		
	Score 2:	16.2 %	10.9 %	3.8 %		
	Score 3:	0 %	10.9 %	6.4 %		
	Score 4:	0 %	26.9 %	21.8 %		
	Score 5:	0 %	13.5 %	15.4 %		
	Score 6:	1.4 %	14.7 %	16.7 %		
	Score 7:	0 %	23.1 %	35.9 %		
p16-Ki67-HMB45 combined score	Score 0:	20.2 %	0 %	N.A.	-	0.987 [0.963–0.997]
	Score 1:	25.7 %	0 %			
	Score 2:	35.1 %	0 %			
	Score 3:	16.2 %	1.3 %			
	Score 4:	1.4 %	13.5 %			
	Score 5:	0 %	13.5 %			
	Score 6:	0 %	26.9 %			
	Score 7:	0 %	16.7 %			
	Score 8:	1.4 %	12.8 %			
	Score 9:	0 %	15.4 %			

### Multiple immunohistochemistry scoring

On the bases of the trends raised analyzing the results of individual Ki-67, p16 and HMB45, we built a scoring system combining the data of these three immunohistochemical markers.

Comparison of the ROC curves of primary melanomas and nevi from the first set of tumours showed a statistically significant greater AUC value using Ki-67 than p16 scoring, this latter being albeit greater but not statistically significantly different from the AUC value obtained with the HMB45 gradient. As a conclusion, we gave greater importance to Ki-67 than p16-scoring and HMB45 gradient scoring in the establishment of our scoring system.

**Fig. 1:**
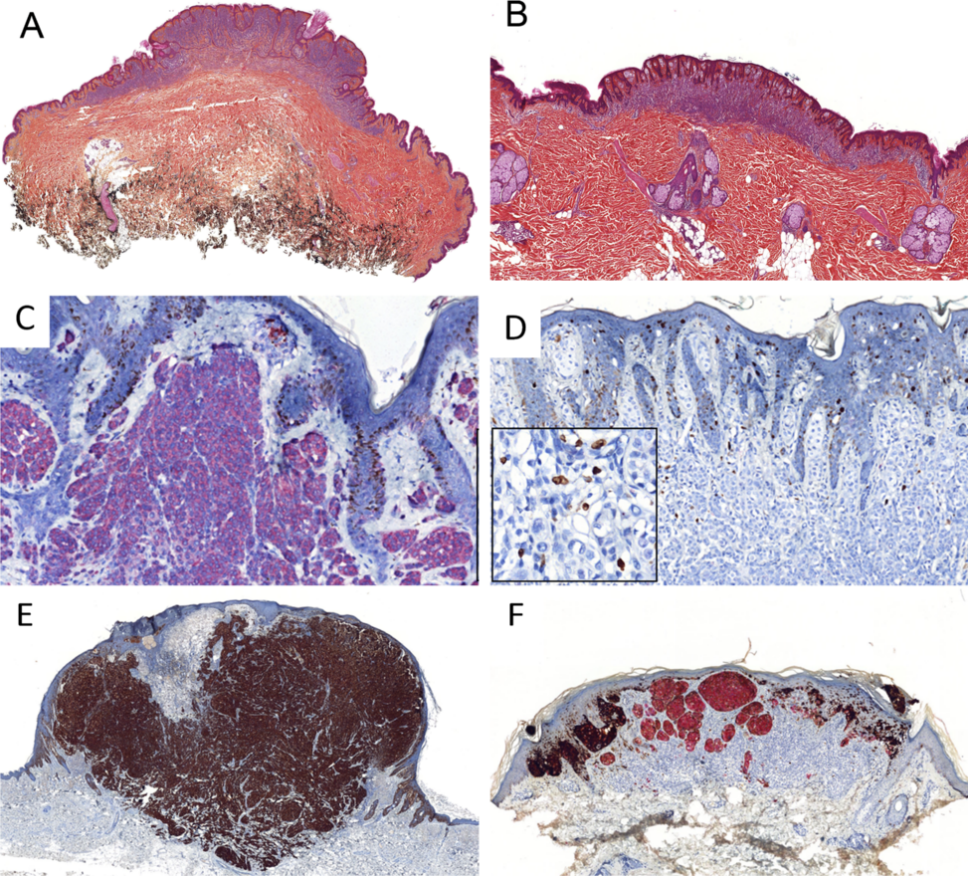
Illustration of immunostaining patterns from the first set of tumours. **a** Dermal nevus in a 39-year-old woman with metastatic melanoma (HES). **b** Second melanocytic tumour of the back in the same patient initially typed as a nevus (HES). **c** The tumour shown in (**a**) presents a strong diffuse red p16 labelling with a Ki-67 index of less than 1 % (see positive control as nuclear DAB staining of basal keratinocytes) (Immunohistochemistry slide). **d** The tumour of the back shown in (**b**) is p16 negative in this field (5 % of stained cells in the whole tumour) and Ki-67 index has been estimated to 8 % of nuclear DAB stained tumour cells; this tumour was finally considered as a nevoid melanoma (the tumour was not stained with HMB45 IHC leading to a p16-Ki-67-HMB45 score of respectively 1 + 3 + 1 = 5). **e** Absence of HMB45 gradient in a nodular melanoma: note the strong and diffuse DAB staining of the whole tumour (HMB45 score 2). **f** HMB45 DAB and MelanA Red double immunostaining of a Spitz nevus: HMB45 staining is limited to junctional component without staining of the dermal nests, here strongly red-stained with anti-MelanA antibody (HMB45 score 0)

The following scoring system was developed to obtain greater values in malignant than in benign tumours. Ki-67 proliferative index was scored using a progressive five-class scale from the less proliferative (score 0) to the more proliferative tumours (score 4): inferior to 2 % (score 0), from 2 to 5 % (score 1), from 6 to 10 % (score 2), from 11 to 20 % (score 3) and superior to 20 % (score 4). The percentage of p16-immunolabeled cells was scored using a four-class digressive scale from score 0 to score 3: expression in more than 50 % of tumour cells (score 0), from 50 to 11 % (score 1), in 10 % or less (score 2), or total absence of p16-expression (score 3). A positive HMB45 gradient was scored 0, a negative scored 2 and the inconclusive scored 1. The scores were finally added to obtain, on the one hand, a p16-Ki-67 score from 0 to 7 and, on the other hand a p16-Ki-67-HMB45 score from 0 to 9. This scoring system is summarized in Table 3 and examples of staining and scoring from the first set of tumours are provided in Fig. 1.

**Table 3:** Proposed three parameters scoring system

Parameter	Score 0	Score 1	Score 2	Score 3	Score 4	Total scores
Ki-67	<2 %	2–5 %	6–10 %	11–20 %	>20 %	p16-Ki-67 score: 0–7 p16-Ki-67-HMB45 score: 0–9
p16	>50 %	11–50 %	1–10 %	0 %	-	
HMB45	Gradient present	Gradient doubtful or inconclusive	Gradient absent	-	-

**Fig. 2:**
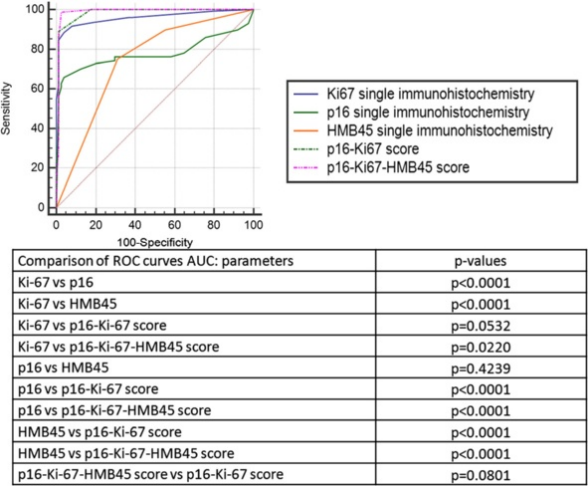
Receiver Operating Characteristic (ROC) curves comparison of single and combined immunohistochemical analyses and *p*-values of the Areas Under the Curves (AUC) of the Receiver Operating Characteristic curves of single and combined immunohistochemical analyses

Comparison of the ROC curves showed the AUC value of this latter p16-Ki-67-HMB45 combination to be significantly superior to individual Ki-67, p16 and HMB45 values (see Fig. 2) but not significantly different from the p16-Ki-67 combined score.

### p16-Ki-67 and p16-Ki-67-HMB45 immunohistochemical scoring systems to differentiate melanomas and nevi

Distribution of the tumours from the first set using different combined scores is summarized in Table 2. Using the p16-Ki-67 scoring system, a score of 2 or 3 had to be considered as threshold values to differentiate nevi and malignant tumours. Nevertheless, many false positive results (i.e., nevi with score equal or higher than 2 or 3) and false negative results (i.e., malignant tumours with score inferior to 2 or 3) are encountered with this scoring system. With a threshold value set at 2 (i.e., a score equal at 2 considered to be related to a malignant tumour), the sensitivity of this test is 100 % but its specificity is 82 %. With a threshold value set at 3, the sensitivity of this test is 91.5 % and its specificity is 98.6 %.

With the p16-Ki-67-HMB45 scoring system, a score of 3 or 4 had to be considered as threshold values. With a threshold value set at 3, the sensitivity of this test is 100 % and its specificity is 81.1 % whereas with a threshold value at 4, the sensitivity of this test is 97.4 % and its specificity 97.3 %.

Finally, in our study, the most efficient test to differentiate benign and malignant melanocytic tumours was the p16-Ki-67-HMB45 combined score with a threshold value at 4 (i.e., a score inferior to 4 in favor of a benign lesion and a score of 4 or higher in favor of a malignant tumour).

In four cases (cases #1 to #4, see Table 4), there was discordance between our scoring results and the initial histopathological diagnosis. Indeed, two tumours (cases #1 and #2) were scored as malignant despite initial benign diagnosis and 2 others (cases #3 and #4) were scored as benign despite initial malignant diagnosis. None of these 4 tumours had known clinical evolution. However, FISH analysis argued in favor of malignant tumours in the 2 initial nevi (cases #1 and #2). IHC slides re-examination of the 2 malignant tumours confirmed the initial diagnosis and concluded in two superficial spreading melanomas developed on pre-existing nevi (cases #3 and #4). These cases and five additional misdiagnosed/difficult tumours (cases #5 to #9) are summarized in Table 4.

**Table 4:** Summary of the challenging tumours

Case & Initial diagnosis	Malignancy criteria	p16 (% & score)	Ki-67 (% & score)	HMB45 gradient (& score)	p16-Ki-67 score	p16-Ki67-HMB45 score
Case #1: Cellular blue nevus, leg, 14 year-old boy	FISH : chromosome 6 polysomy, 8p34 gain, 9p21 double deletion, 11q13.1 gain	15 % (score 1)	3 % (score 1)	Absent (score 2)	Score 2	Score 4
Case #2: Spitz nevus, back, 42 year-old woman	FISH: chromosomes 6 and 8 polysomies, 9p21 double deletion, 11q13.1 gain	0 % (score 3)	12 % (score 3)	Absent (score 2)	Score 6	Score 8
Case #3: SSM on preexisting nevus, back, 59 year-old man	Clear SSM features	Whole lesion : 70 % (score 0) Atypical component : 0 % (score 3)	10 % (score 2)	Doubtful (score 1)	Whole lesion: score 3 SSM: score 6	Whole lesion: score 2 SSM: score 5
Case #4: SSM on preexisting nevus, ear, 64 year-old man	Clear SSM features	Whole lesion : 70 % (score 0) Atypical component : 0 % (score 3)	10 % (score 2)	Doubtful (score 1)	Whole lesion: score 3 SSM: score 6	Whole lesion: score 2 SSM: score 5
Case #5: Nevus, back, 41 year-old man	Metastatic evolution : reclassed as SSM	80 % (score 0)	20 % (score 3)	Absent (score 2)	Score 3	Score 5
Case #6: Nevus, back, 39 year-old woman	Metastatic evolution : reclassed as nevoid melanoma	5 % (score 2)	8 % (score 2)	Inconclusive (score 1)	Score 4	Score 5
Case #7: Atypical nevus, knee, 43 year-old man	Metastatic evolution : reclassed as nodular melanoma	0 % (score 3)	16 % (score 3)	Present (score 0)	Score 6	Score 6
Case #8: Spitz nevus, thigh, 24 year-old man	Metastatic evolution : reclassed as Spitzoid melanoma	0 % (score 3)	12 % (score 3)	Absent (score 2)	Score 6	Score 8
Case #9: Nevoid melanoma, 17-year old girl, calf	Concomitant metastasis	20 % (score 1)	12 % (score 3)	Absent (score 2)	Score 4	Score 6
^a^Case #10: Spitzoid tumour, knee, 17-year old girl, knee	Review by an international expert in melanocytic pathology, reclassed as Spitzoid melanoma	15 % (score 1)	25 % (score 4)	Inconclusive (score 1)	Score 5	Score 6
^a^Case #11: SSM, back, 58-year old woman	No final histological criteria of malignancy, reclassed as a compound nevus	60 % (score 0)	1 % (score 0)	Inconclusive (score 1)	Score 0	Score 1

*SSM* superficial spreading melanoma

^a^cases from the validation set

### Analyses of the validation set of tumours

**Fig. 3:**
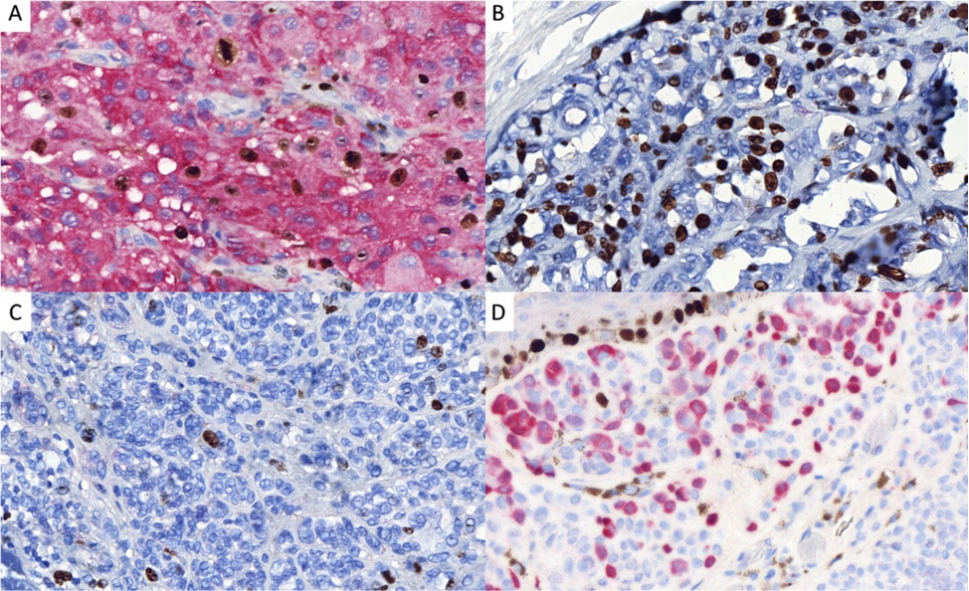
Illustrations of some Ki-67 (DAB) and p16 (*Red*) immunostaining results of the validation set of tumours (×400). **a** Diffuse p16 staining (score 0) within a melanoma with a Ki-67 index of about 15 % (score 3). **b** p16 negativity (score 3) within a melanoma with a Ki-67 index estimated at 50 % (score 4). **c** p16 negativity (score 3) within a melanoma with a Ki-67 index estimated at 3 % (score 1). D: About 40 % of nevocytes are stained with p16 (score 1) within a dermal nevus with a Ki-67 index of 1 % (note the Ki-67 stained basal keratinocytes as internal positive controls)

**Fig. 4:**
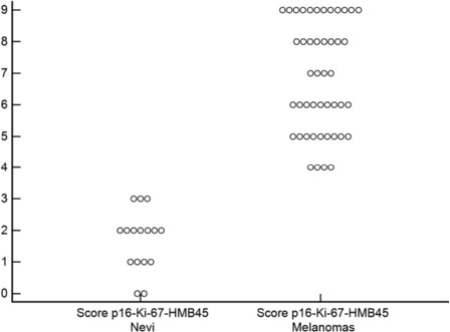
Summary of p16-Ki-67-HMB45 combined scores of the nevi and melanomas in the validation set of tumours

The 46 malignant melanomas and 16 nevi of the validation set of tumours were rightly classified as benign or malignant tumours using the p16-Ki-67-HMB45 combined score (see Figs. 3 and 4). Note that the spizoid tumour classified by an expert as a spitzoid melanoma had a score of 6 (case #10, see Table 4) and the tumour that was initially misclassified as a SSM and finally considered as a compound nevus had a score of 1 (case #11, see Table 4).

## Discussion

Taking into account not only the frequency of melanocytic tumours in daily practice of surgical pathology but also the issues and poor inter-observer reproducibility to classify some melanocytic tumours as benign nevi or malignant melanomas, many molecular and immunohistochemical attempts have been made to find a clear-cut criterion to distinguish nevi from melanoma in case of histologically difficult tumour. Nevertheless, molecular methods are expensive and require highly skilled pathologist and no immunomarker has been considered sensitive and specific enough in this field. Histopathological examination is still the gold standard to classify the melanocytic tumours. Most of the immunohistochemical studies have focused on the search of a single marker that can discriminate between nevi and melanomas. Ki-67, HMB45 and p16 are some of these markers, reported with various but finally always limited performances.

Ki-67 index was reported to be higher in malignant melanomas than in benign nevi. Its expression has also been correlated to prognosis in patients with melanomas [18]. Nevertheless, there is a great heterogeneity in the IHC analysis strategy regarding techniques, count methods (manual or automatic, single or double staining, number of tumour cells nuclei analyzed, mean value calculated in the whole lesion or only in the most proliferative hotspots, quantitative or semi-quantitative approach with various scales). Different thresholds have been evaluated in an attempt to find a cut-off between nevi and melanoma, with final proposed cut-offs of 2 to 10 %. Nevertheless, in all studies, melanomas with low Ki-67 index below the cut-off values are reported [11, 19–29]. Although a strong Ki-67 index is an argument for a malignant lesion, a low Ki-67 index does not eliminate a melanoma. Ki-67 emerged as the most efficient marker in our study to distinguish melanomas and nevi but the same limitations appeared concerning low-proliferative melanomas.

HMB45 immunostaining has also been considered as a helpful tool to distinguish benign from malignant melanocytic tumours. The contribution of this IHC technique consisted in looking for a gradient of expression that is preserved in a benign lesion and abolished in a malignant lesion [14, 30–33]. Nevertheless, sensitivity from 69 to 93 % of HMB45 staining in melanomas is reported [29]. Moreover, there is no established positive predictive value for HMB-45 staining in the diagnosis of nevoid melanoma; also some nevi (i.e., deep penetrating nevi, blue nevi and fibrotic dysplastic nevi) show diffuse (i.e., non-gradient) expression of HMB-45 from top to bottom [14, 34].

Finally, expression of p16 has also been widely studied in its ability to distinguish melanomas and nevi with conflicting reports and very heterogeneous data, probably due to the same limitations as mentioned in Ki-67 scoring. A general trend is the highly conserved p16 expression in benign nevi and the frequent loss in melanomas. Nevertheless, many melanomas still express p16. Even if a p16-negative profile is more frequent in melanomas, a p16-positive profile does not allow concluding in a benign or malignant tumour. The definition of a p16-negative or positive melanocytic lesion is by itself unclear and not consensual in the different studies. Some have considered different intensities, percentage, or localization (i.e., nuclear and/or cytoplasmic expression) of staining to qualify a lesion as p16-positive or negative. Nevertheless, none of these criteria appear discriminative enough and the utility of this marker solely does not seem pertinent, as previously reported [16, 35–43].

Only few studies have searched for a scoring system combining these useful but imperfect tools. Ki-67 and p16 have been combined in some studies, pointing out not only an increase of the proliferative index in malignant tumours but an inverse relationship between Ki-67 and p16 with a Ki-67 index higher in p16-weak or -negative tumours than in p16-positive tumours [16, 37, 44, 45]. Ki-67 and HMB45 have also been combined in other studies pointing out an improvement in the distinction between melanoma and nevus compared to single marker scorings; nevertheless, 10 of 78 nevi were misclassified using this combined approach in one study [14, 33].

Taken individually, we demonstrated poor performances of HMB45 and p16 IHC to distinguish between melanomas and nevi. Ki-67 was the most efficient single IHC but remained imperfect because of some low proliferative malignant tumors. As a consequence, we chose to combine the most efficient (Ki-67) and one (p16) or two (p16 and HMB45) IHC to search for improving distinction between malignant and benign lesions. Moreover, Ki-67 is the sole marker with nuclear-restricted staining while p16 staining is cytoplasmic and nuclear and HMB45 has a cytoplasmic staining. We chose to combine p16 and Ki67 in a double-stain IHC instead of combining p16 with HMB45 because the combination of p16 and HMB45 would have impaired the interpretation of these two markers because of overlapping cytoplasmic staining.

As our aim was to propose a score in which high values would be more related to malignancy and low values to a more benign melanocytic tumor, the best performances were achieved with p16-Ki-67-HMB45 scoring system that appeared valuable in our two sets of tumours. Further analyses argued for possible malignant tumours concerning two tumours initially considered as nevi (cases #1 and #2). In two SSM arising in preexisting nevi (cases #3 and #4), the p16-Ki-67 IHC helped to distinguish between the malignant and benign components. Our score also seems able to detect some challenging tumours as nevoid melanomas and misdiagnosed tumours with known metastatic evolution (cases #5 to #11).

## Conclusion

To conclude, we have built a valuable triple p16-Ki-67-HMB45 immunohistochemistry scoring system to help pathologists in the differential diagnosis of melanomas and nevi. As our score seems able to type some challenging tumours, the results of our diagnostic study have to be confirmed in other independent studies notably about difficult and so-called “ambiguous” melanocytic tumours referred to dermatopathologists expert in melanocytic pathology. Additional studies are also necessary to confront our score to clinical evolution to explore its potential prognostic significance.
